# Class III treatment with mini-implants anchorage in young adult patients: short and long-term results

**DOI:** 10.1590/2177-6709.28.2.e23spe2

**Published:** 2023-06-05

**Authors:** Giuliano Bortolo MAINO, Giovanna MAINO, Francesca CREMONINI, Luca LOMBARDO

**Affiliations:** 1 University of Ferrara, Postgraduate School of Orthodontics (Ferrara, Italy).

**Keywords:** Skeletal anchorage, Rapid palatal expander, Alt-RAMEC protocol, Class III malocclusion, Adult patients

## Abstract

**Introduction:**

Class III malocclusion should be intercepted and treated at early age, to prevent the necessity of future complex and expensive procedures. The orthopedic facemask therapy has the goal to achieve skeletal changes, minimizing side effects on dentition. The use of skeletal anchorage, combined with Alternate Rapid Maxillary Expansion and Constriction (Alt-RAMEC) protocol, may be effective in treating a greater number of growing Class III patients.

**Objective:**

To summarize the existing evidence-based literature on Class III malocclusion treatment in young adult patients, and to illustrate its application and effectiveness, by presenting an emblematic case report.

**Conclusion:**

The resolution of the present case, its long-term follow up, along with the studies conducted on a larger sample, demonstrate the effectiveness of the strategic combination of orthopedic and orthodontic treatments by using an hybrid rapid palatal expander and Alt-RAMEC protocol for treating Class III malocclusions in adult patients.

## RESUMO

**Introdução:**

A má oclusão de Classe III deve ser interceptada e tratada em idade precoce, a fim de evitar uma futura necessidade de procedimentos complexos e invasivos. O tratamento com máscara facial ortopédica tem o objetivo de obter alterações esqueléticas, minimizando os efeitos colaterais na dentição. O uso de ancoragem óssea em mini-implantes, associada ao protocolo Alt-RAMEC (*Alternate Rapid Maxillary Expansion and Constriction*) pode ser eficaz no tratamento de um grande número de pacientes Classe III em crescimento.

**Objetivo:**

Realizar uma síntese da literatura baseada em evidência sobre o tratamento da má oclusão de Classe III em pacientes adultos jovens, e ilustrar sua aplicação e eficácia por meio do relato de um caso emblemático.

**Conclusão:**

A resolução e o acompanhamento em longo prazo do caso apresentado, juntamente com estudos conduzidos em uma amostra maior, demonstram a eficácia da combinação estratégica dos tratamentos ortopédico e ortodôntico usando um expansor palatal híbrido e o protocolo Alt-RAMEC para corrigir a má oclusão de Classe III em pacientes adultos.

## CLASS III SKELETAL MALOCCLUSION

Class III skeletal malocclusion was first defined by Angle in 1899, based on the sagittal ratio of the permanent first molars and, subsequently, the growth pattern. Class III is characterized by a more advanced position of the mandible compared to the maxilla, which can also be associated with a more mesial position of the permanent mandibular first molar. Indeed, although Class III is often considered synonymous of mandibular prognathism, though present in many individuals, a prognathic mandible turns out to be only one of the many components that characterize this complex malocclusion. In fact, Guyer et al.^1^ found that the most common situation in Class III, seen in about 30% of cases, is a combination of two factors: mandibular prognathism and maxillary retrusion. In their investigation, only 19.5% of the subjects had maxillary retrusion with normal mandibular prominence, and 19.1% presented normal maxilla with mandibular protrusion.

The aetiology of Class III malocclusion is multifactorial and rather complex, encompassing environmental, functional and genetic factors. However, the genetic transmission of the tendency to mandibular prognathism is the main cause underlying the aetiology of Class III. The prevalence of Class III malocclusion varies significantly between populations of different ethnicities: Southeast Asia in particular has the highest prevalence, up to 15.8%; while Europe has the lowest, around 1.2%.^2^ Interestingly, McGuigan’s^3^ study of the orthodontic records of 40 members of the House of Habsburg royal family showed that 33 of them had a Class III intermaxillary relationship characterized by mandibular prognathism.

Some important longitudinal studies have been conducted with the purpose of studying the course of craniofacial changes occurring during growth in subjects with untreated skeletal Class III malocclusion. To this end, skeletal changes were characterized using cephalometric tracings, and compared with subjects with a Class I intermaxillary relationship.^4^ By means of the cervical vertebral maturation method (CVM), it was shown that the growth peak in Class III subjects occurs on average at around 10-12 years for females and 12-15 years for males. The mandible length increases substantially in this age range, especially in male subjects, by more than 3 mm per year. This observation is entirely in line with the findings of Baccetti et al.^5^, that the growth peak in Class III is more extensive and marked than normal. In addition to that, in Class III subjects the residual mandibular growth after the pubertal peak is particularly pronounced. In female subjects older than 13 years and male subjects older than 15 years, the mandibular length continued to increase annually by 1.5-2 mm up to the age of about 17 years in both groups.^4^ This marked worsening of relative mandibular prognathism with growth is accompanied by incremental dental movements, in order to compensate for the discrepancy between the jaw bones.

## ORTHOPEDIC TREATMENT: RAPID PALATAL EXPANDER + FACEMASK

Intercepting this malocclusion at an early age and implementing orthopedic treatment is still the gold standard therapeutic choice to achieve significant improvement in both skeletal and dental parameters. Early treatments that have been widely documented and studied in the literature include the facemask, chin guard, functional appliances such as Frankel III and Bionator III, and the reverse Twin-Block.^6^ ^,^ ^7^ The most recent meta-analyses show that maxillary protraction using a facemask (FM) represents the most effective therapeutic solution for Class III correction, achieving major dental and skeletal effects in the short term,^8^ ^,^ ^9^ which can be summarized as clockwise mandibular rotation and protraction of the maxilla. The clockwise mandibular rotation of about 1.5° causes a corresponding retraction and lowering of point B, with partial resolution of the sagittal discrepancy.^9^ The beneficial effects of the FM are therefore mainly attributable to an advancement of the maxilla, rather than to a real inhibition of mandibular growth, which is largely only redirected. In particular, the FM can achieve a maximum maxillary advancement of 4 mm, but this orthopedic effect tends to decrease as the patient ages.^9^

Sagittal correction should always be preceded by normalization of the maxillary transverse dimensions, which in Class III skeletal malocclusion appear to be reduced in the presence of a hypoplastic maxilla. In this regard, the beneficial effects of a treatment featuring rapid maxillary expansion (RME) in combination with FM are widely documented in literature, especially with regard to the disarticulation of the circummaxillary sutures, enabling protraction of the maxilla.^10^ To maximize the effect of FM, a very different and specific expansion protocol, the Alternate Rapid Maxillary Expansion and Constriction (Alt-RAMEC), was introduced by Liou and Tsai^11^ in 2005. This involves a week of expansion (four daily activations for a total of 1 mm of expansion) alternating with a week of contraction (four daily activations for a total of 1 mm of contraction) for nine consecutive weeks, so as to mobilize the sutures and the maxilla. This phase is followed by a series of expansion activations via the standard protocol, until overcorrection of the transverse dimensions is achieved.^11^ ^,^ ^12^

Application of the Alt-RAMEC protocol leads to more extensive opening of the circummaxillary sutures than the traditional expansion protocol.^13^ Its efficacy was first demonstrated in patients with cleft lip and palate, but many clinicians now use it to increase the effectiveness of FM therapy for Class III malocclusion in adolescent patients. Indeed, as demonstrated by a systematic review with meta-analysis conducted by Foersch et al.,^14^ combined with FM, the Alt-RAMEC protocol can lead to more pronounced skeletal effects in the treatment of patients with maxillary deficiency when applied in the prepubertal phase.^15^ ^,^ ^16^

## ORTHOPEDIC TREATMENT: SKAR III PROTOCOL

The goal of orthopedic FM therapy is to achieve true skeletal changes, minimizing adverse effects on the dentition. Considering the age of sutural ossification, the effectiveness of the combined RME + FM treatment is greatest when patients are treated at a very early age. Several studies have shown that if dental anchorage is used alone, the adverse effects will be more accentuated as the growth peak and complete ossification of the sutures approaches. Even when associated with the Alt-RAMEC protocol, traditional RME + FM produces undesirable effects on the dentition - in particular, excessive mesial inclination and extrusion of the maxillary molars, excessive proclination of the maxillary incisors, and an increase in lower facial height, with clockwise rotation of the mandibular plane.^17^ ^,^ ^18^

To counter this tendency, a series of skeletal anchorage systems have been devised in recent years, and used to effectively treat a greater number of growing Class III patients, with minimal dentoalveolar effects. For example, in 2018, Maino et al.^19^ published a study on skeletal and dental changes observed in a sample of patients treated with a simplified and standardized protocol, featuring the FM and the Alt-RAMEC protocol applied to a Hyrax-type hybrid rapid palatal expander (h-RPE). This innovative device was given the name of SkAR III (Skeletal Alt-RAMEC for Class III), and it combines dental anchorage, by means of two bands positioned on the first maxillary molars, and skeletal anchorage on two palatal mini-implants.

### SHORT-TERM RESULTS

**Figure 1:**
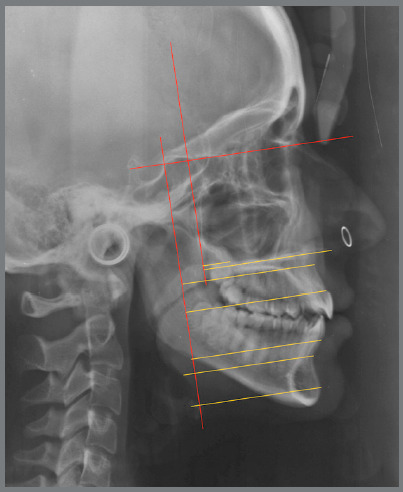
Cephalometric analysis performed according to the Baccetti et al.^17^ and DeClerck et al.^33^ method.

Despite the great difficulties in preventing residual mandibular growth, there is good scientific evidence that early treatment with the SkAR III protocol can reduce the need for orthognathic surgery in adulthood, or at least reduce the amount of surgical correction needed, resulting in more predictable and stable results over time.^20^ ^,^ ^21^ In a study with 27 patients (15 females, 12 males; mean age: 11.3 ± 2.5 years) treated using the same protocol^19^ (i.e., SkAR III followed by four months of FM protraction), point A advanced on average by 3.4 mm, as compared to the vertical reference plane (VertT) (Fig 1), and presented a statistically significant alteration. On the other hand, point B remained relatively stable, and there was 0.22 mm of pogonion (Pg) advancement on average. This led to a 2.5° increase in the SNA angle and a significant improvement in the sagittal relationship, with an increase of 13.4° on average in the ANB angle and 14.92 mm in the Wits appraisal. 

**Figure 2:**
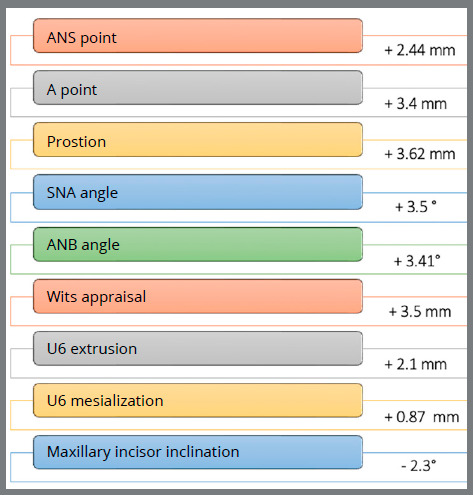
Short-term outcomes, derived from Maino et al.^19^

As regards adverse effects, on the vertical plane there was a slight increase of facial divergence, of only 1.64° at the end of treatment. As for dentoalveolar changes, there was 0.42-mm extrusion of the maxillary first molar in relation to the palatal plane, and mesial inclination of 0.87 mm in relation to VertT. The maxillary incisor, on the other hand, underwent a retroclination of 2.26° in relation to the palatal plane, with an average inclination reduction from 110° to 107.9°. These short-term results also led to a general improvement in facial aesthetics, with a positive psychological impact during adolescence (Fig 2).

More importantly, however, nearly seven years after the end of the orthopedic phase, 27 of the 28 patients had non-extraction orthodontic treatment with fixed multibracket appliances to correct the alignment, levelling and coordination of the arches. Skeletal and dental measurements were performed on laterolateral radiographs taken after an average follow-up period of 7 years, and compared with the lateral radiographs taken before and after the orthopedic treatment with SkAR III + FM. 

### LONG-TERM RESULTS

Due to residual mandibular growth, treatment stability poses a major challenge in Class III patients, and is often associated with a high rate of relapse. A recent systematic review with meta-analysis reported that the anteroposterior benefits of RME + FM gradually decrease over time, the effectiveness of maxillary protraction being particularly poor at follow-up assessments more than three years on.^22^ The overall worsening of cephalometric values at long-term follow-up is due to the fact that the growth peak in Class III subjects is delayed and more pronounced than in Class I subjects.

**Figure 3:**
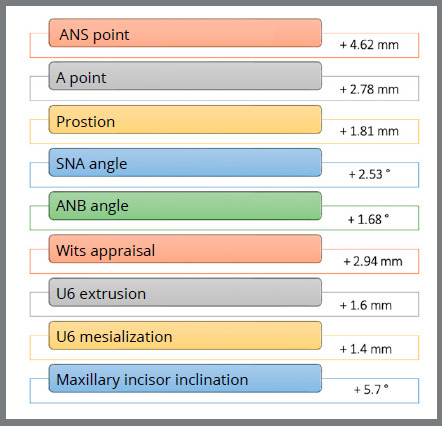
Long-term outcomes, from the start of treatment to follow-up (average interval 7 years, 10 months).

**Figure 4:**
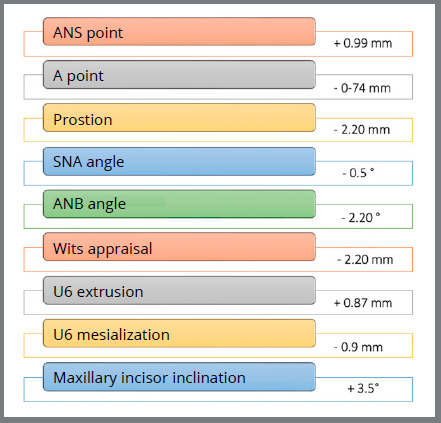
Changes from the end of orthopedic treatment to long-term follow-up.

Nevertheless, the measurements made on lateral radiographs at the beginning of orthopedic treatment (average age 11 years, 4 months) and at follow-up (average age 19 years, 10 months) in a sample of 27 patients showed the orthopedic benefit effect of the SkAR III, Alt-RAMEC and FM protocol (Fig 3).^23^ The results demonstrated the long-term effectiveness and overall stability of the approach used. Although the end-of-growth assessment indicated that some relapse has occurred (Fig 4), the ANB and Wits values remained significantly improved seven years after the end of treatment. Even considering the slight relapse recorded over time (0.7 mm in 7 years), the overall advancement of point A was 3 mm. No statistically significant long-term growth was evidenced at either Pg or B points, meaning that most mandibular growth had already taken place during the orthopedic treatment, which was performed at a later age in most patients.

According to Eslami et al.,^24^ a Wits value within -5.8 mm can be effectively treated or camouflaged, while more negative values should ideally indicate orthognathic surgery. At end-of-growth follow-up, only one patient from the entire sample required orthognathic surgery (Wits: - 14.80 mm). All other patients were successfully treated using orthodontic camouflage. To illustrate the extent to which orthodontics can be used to resolve residual issues in Class III cases, a very complex case treated by SkAR III followed by fixed appliances and intermaxillary auxiliaries will now be described.

### EXAMPLE CLINICAL CASE

**Figure 5:**
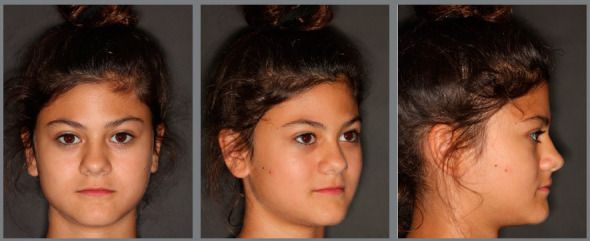
Pretreatment extraoral photographs.

**Figure 6:**
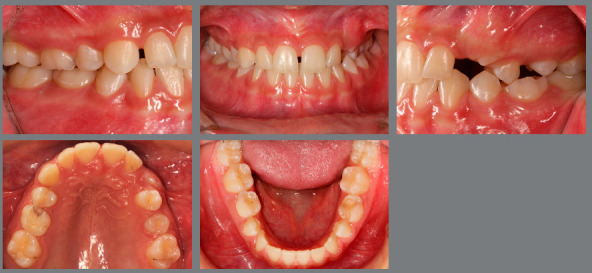
Pretreatment intraoral photographs.

The patient came to our attention at the age of 14 years. Extraoral analysis (Fig 5) revealed a deficit of the middle third of face, a particularly concave profile and an accentuated chin. There was also a slight mandibular deviation to the left. Intraoral analysis (Fig 6) showed a late tooth eruption relative to age, with second deciduous molars still present. On the transverse plane, the maxilla displayed contraction, and the lower Wilson curve was increased. On the vertical plane, the overbite was increased. Although the molar ratio was Class I, there was already dental compensation for the skeletal malocclusion at the level of the incisors, with the mandibular incisors being lingually inclined. Black corridors were present at the smile.

**Figure 7:**
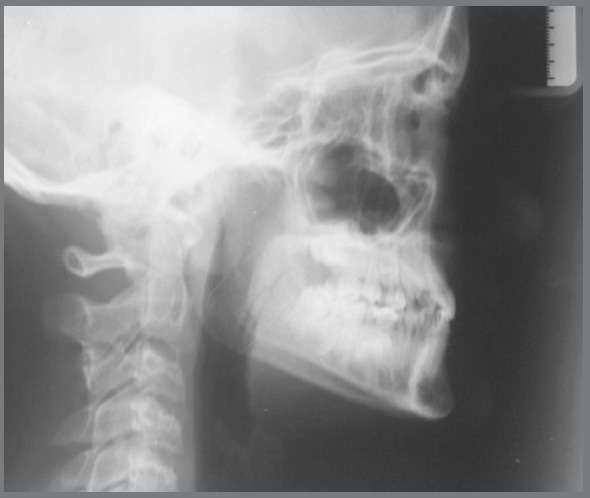
Pretreatment lateral radiograph.

Cephalometric analysis performed on the initial lateral radiograph (Fig 7) revealed a skeletal Class III (ANB: -1.5°, Wits appraisal: -4.3 mm), with a hypodivergent facial growth pattern (FMA: 18°). In addition, the dentoalveolar compensation of the sagittal discrepancy was confirmed, with mandibular incisors retroclination (IMPA: 73°).

Considering the family history and the Class III growth pattern, one option was to wait until the craniofacial growth was completed, and then re-evaluate the case with a view to offering either orthodontic treatment or a surgical-orthodontic approach - if the skeletal Class III was excessively severe. However, the parents asked for an orthodontic intervention to improve the face concavity, which they said was worsening. In order to maximize skeletal outcomes, and considering the age of the patient, the proposed therapeutic option was to use the SkAR III protocol to actively correct the Class III skeletal discrepancy, increasing the forward displacement of the upper jaw and avoid worsening dental compensation. This would counteract the subsequent Class III growth trend, allowing for the second orthodontic phase once permanent dentition was completed. 

**Figure 8:**
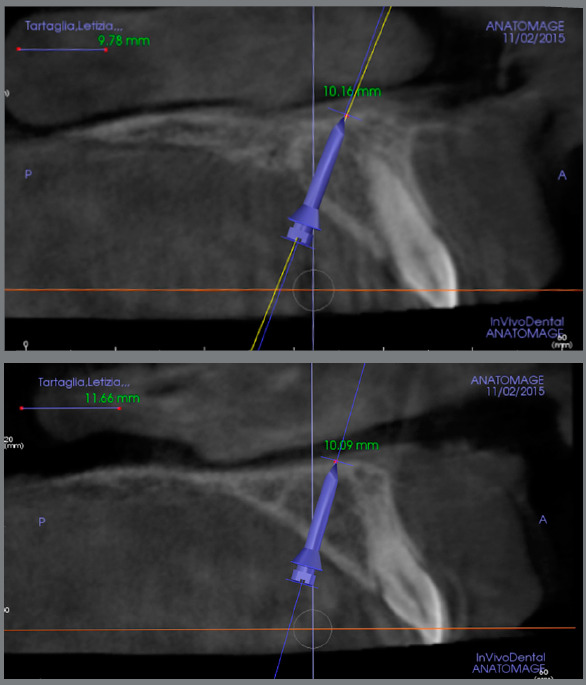
Digital planning of the mini-implants insertion by matching the DICOM files of the CBCT and the STL files of the digital models, according to the MAPA protocol.

Due to family reasons, the treatment started 8 months later. The mini-implants insertion was determined using digital planning, matching the DICOM files of the CBCT and the STL files of the digital models, according to MAPA (MAino-PAoletto) protocol.^19,20,25^ After a careful evaluation of the amount of bone availability in the palate, the ideal direction, position and length of the two mini-implants to be inserted were defined, with the purpose of obtaining a bicortical contact, which, according to the literature, would increase primary stability and consequently the mini-implants success rate^25^ ^-^ ^27^ (Fig 8). Two mini-implants (2-mm diameter and 11-mm length, K2, Konic Spider Screw, HDC, Thiene Italy) were selected, and a surgical guide digitally designed and printed was used to facilitate the mini-implants insertion and improve their predictability.^28^ ^,^ ^29^

**Figure 9:**
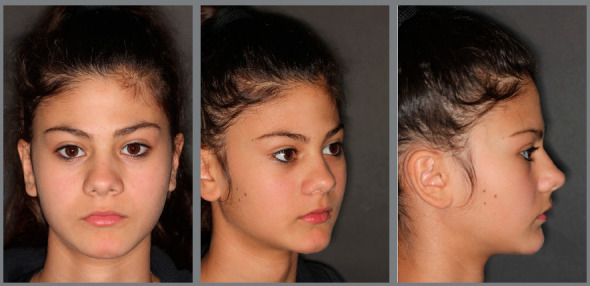
Extraoral photographs after the first orthopedic phase.

**Figure 10:**
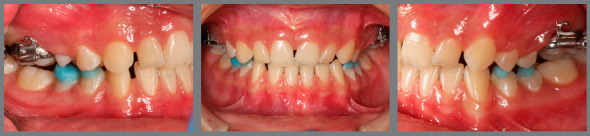
Intraoral photographs after the first orthopedic phase.

**Figure 11:**
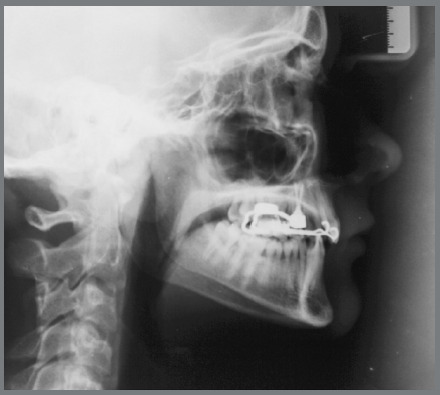
Lateral radiograph after first orthopedic phase.

Precise and accurate digital planning allowed for the installation of the orthodontic appliance in the same session in which the mini-implants were inserted (MAPA-One Visit Protocol).^30^ At the end of the facemask phase (4 months), at 15 years of age, the profile appeared convex, and there was appreciably greater maxillary support, with advanced projection of the upper lip (Fig 9). Intraoral photographs showed the overcorrection achieved for both the transversal and sagittal discrepancy (Fig 10), with a Class II molar and canine relationship. The cephalometric values (Table 1) obtained from the radiograph taken with the SkAR III still installed (Fig 11) revealed the great improvement in the sagittal ratio between the jaws (ANB modified from -2° to 5°, Wits from -3 mm to 3.5 mm). 

**Table 1:** Pretreatment and posttreatment cephalometric measurements.

	Normal	Initial	End of facemask	End of treatment	Long term follow-up
SNA (degrees)	82°	85°	90°	89.3°	88.9°
SNB (degrees)	80°	87°	85°	85.3°	86.7°
ANB (degrees)	2°	-2°	5°	4°	2.2°
SN.GoGn (degrees)	32°	25°	27°	21.0°	20.8°
Occl.SN (degrees)	14°	9°	5.6°	27°	23°
1-NA (mm)	4	4mm	-1,5	-0,5	3,0 mm
1.NA (degrees)	22°	26°	12.2°	21.0°	29.3°
1-NB (mm)	4	1mm	-2,2	1,7 mm	2.1 mm
1.NB (degrees)	25°	9°	4.2°	21.2°	20.7°
Po-NB (mm)	-	4mm	3,8 mm	4.0 mm	3.0 mm
1/1 (degrees)	131°	145°	159.1°	133.6°	127.9°
Wits appraisal	-	-3mm	+3,5mm	+2mm	+1mm
SN.PP (degrees)	8°	4°	1.7°	3.6°	1.5°
PP.GoGn (degrees)	25°	20°	23.0°	21.0°	20.8°
PP.1 (degrees)	110°	106°	103.5°	114.0°	119.6°
Overjet (mm)	3,5	3 mm	7 mm	6 mm	4 mm
Overbite (mm)	2	2,5mm	4.2 mm	2.4 mm	3.0 mm
FMIA (degrees)	65°	85°	85.4°	69.1°	69.5°
FMA (degrees)	25 /27°	18°	22.2°	19.4°	20.5°
IMPA (degrees)	90°	73°	72°	91.5°	89.9°
Sn (mm)	-	68 mm	68 mm	68 mm	68 mm
GoGn (mm)	-	79 mm	80 mm	81 mm	81 mm
AN.Pg (degrees)	-	-3°	+3°	+1°	+1°

**Figure 12:**
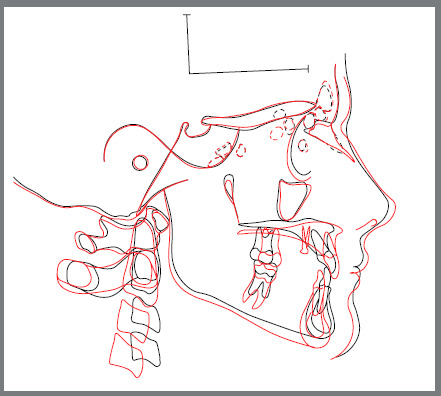
Pretreatment (black) and post-treatment (red) cephalometric tracings superimposition on the anterior cranial base, according to “The Structural Method” developed by Bjork^32^.

Possible adverse effects from maxillary expansion and protraction were contained. In particular, there was just slight increase in mandibular divergence. The extent of the maxillary advancement can be seen in the cephalometric tracings superimposition performed on the anterior cranial base (Fig 12). The total treatment time was four months, then the appliance was removed, and the patient was scheduled for long-term monitoring.

**Figure 13:**
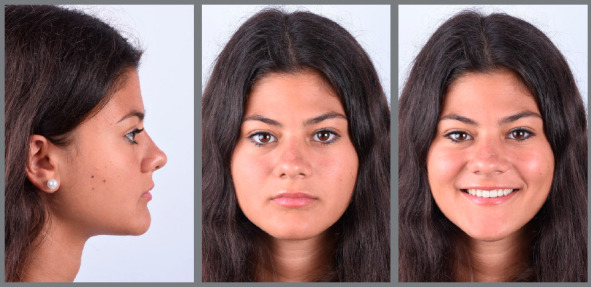
Extraoral photographs at the end of orthodontic treatment.

At the age of 20 years, about 6 years after the end of the orthopedic phase of Class III correction, no profile worsening or alteration was evident (Fig 13).

**Figure 14:**
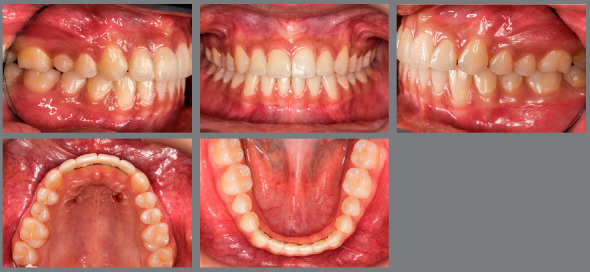
Intraoral photographs at the end of orthodontic treatment.

**Figure 15:**
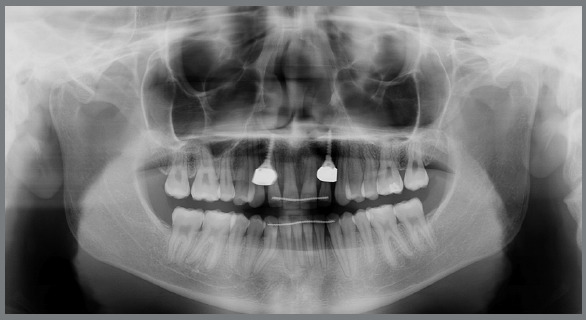
Panoramic radiograph at the end of orthodontic treatment.

After the second phase of orthodontic treatment, aiming at normalizing all the alignment parameters, the intraoral assessment (Fig 14) revealed perfect Class I molar and canine intercuspidation, correctly centered midlines, and good transverse dimensions. Upper and lower splints were fixed, and a Essix appliance was delivered for night wear, with retention purposes. The panoramic radiograph evidenced good root parallelism (Fig 15).

**Figure 16:**
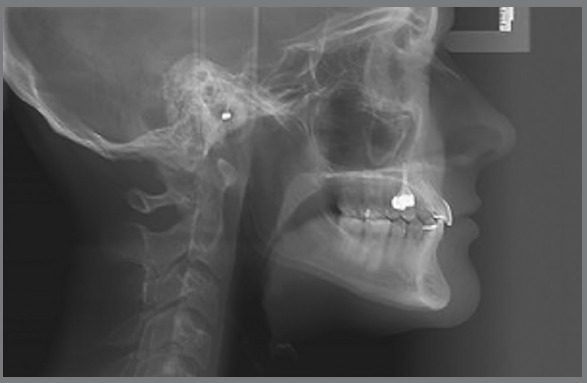
Lateral radiograph at the end of orthodontic treatment.

**Figure 17:**
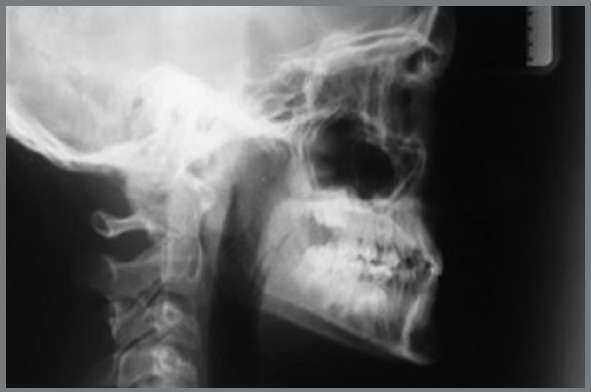
Lateral radiograph at 2-year follow-up.

Comparative cephalometric analysis of the final radiograph (Fig 16) and the previous ones showed slight relapse (ANB from 5.0° to 4.0°, Wits from 3.5 mm to 2 mm), probably due to the post-pubertal residual growth of the mandibula and less growth of the maxilla.^5^ The orthodontic treatment implemented in the second phase by using the Bidimensional Technique^31^ aimed to keep the mandibular incisors as far forward as possible and mesialize premolars and molars to provide better support for the lips. New latero-lateral radiograph after two years from the end of orthodontic treatment was performed, and the cephalometric values confirmed good stability of the final results (Fig 17).

## CONCLUSION

The resolution of the present case and the studies conducted on a larger sample^19^ of patients demonstrate the effectiveness of the strategic combination of orthopedic and orthodontic treatments by using mini-implants, in the resolution of moderate to severe Class III skeletal malocclusions. The combined use of hybrid palatal expander and the Alt-RAMEC protocol, followed by maxillary protraction with facemask, successfully corrected skeletal Class III due to substantial maxillary advancement, with almost no adverse dental effects, even in adolescent patients.
